# Transformation of the rodent malaria parasite *Plasmodium chabaudi *and generation of a stable fluorescent line PcGFP_CON_

**DOI:** 10.1186/1475-2875-7-183

**Published:** 2008-09-22

**Authors:** Sarah E Reece, Joanne Thompson

**Affiliations:** 1 Institute of Immunology and Infection Research, School of Biological Sciences, University of Edinburgh, UK; 2 Institute of Evolutionary Biology, School of Biological Sciences, University of Edinburgh, UK

## Abstract

**Background:**

The rodent malaria parasite *Plasmodium chabaudi *has proven of great value in the analysis of fundamental aspects of host-parasite-vector interactions implicated in disease pathology and parasite evolutionary ecology. However, the lack of gene modification technologies for this model has precluded more direct functional studies.

**Methods:**

The development of *in vitro *culture methods to yield *P. chabaudi *schizonts for transfection and conditions for genetic modification of this rodent malaria model are reported.

**Results:**

Independent *P. chabaudi *gene-integrant lines that constitutively express high levels of green fluorescent protein throughout their life cycle have been generated.

**Conclusion:**

Genetic modification of *P. chabaudi *is now possible. The production of genetically distinct reference lines offers substantial advances to our understanding of malaria parasite biology, especially interactions with the immune system during chronic infection.

## Background

The development of transfection technologies in *Plasmodium *have led to substantial advances in malaria research [1-3]. The first successful transient transfection to be reported was performed in *Plasmodium gallinaceum*, followed by *Plasmodium falciparum *[4,5]. However, stable gene disruption and replacement technologies were developed first for the rodent malaria parasite, *Plasmodium berghei *[3,6-9], and have subsequently proved particularly useful in analysis of proteins mediating sexual development and transmission of the parasite through the mosquito vector [10-13]. Stable transformation has also been achieved for *Plasmodium yoelii *[14] but *P. berghei *has been the focus for the development of technical advances in transfection [2].

However, *P. berghei *causes a rapid and virulent infection leading to widespread tissue pathology and early death without effective host immune control making the investigation of host adaptive immune responses and chronic malaria infections challenging [15,16]. The lack of genetically distinct *P. berghei *clones limits its use as a model for investigations of the evolution and ecology of host-parasite interactions [17,18]. Furthermore, transmission of *P. berghei *through the mosquito vector occurs at lower temperatures (18–21°C) and is longer (21 days) than transmission of human or other rodent malaria parasites (> 24°C for 10–14 days) and *P. falciparum*. This may influence studies of parasite biology and development in the vector as parasite-vector interactions are sensitive to temperature (e.g. [19]).

In contrast, the rodent malaria parasite *P. chabaudi *could provide a more relevant model for investigating anti-malarial host immune responses because infections are usually controlled by host immunity (reviewed in [16,20,21]). *Plasmodium chabaudi *also shares a number of life history features with the most severe human malaria parasite, *P. falciparum*, including mature erythrocyte preference, synchronous schizogony, sequestration, rosetting, antigenic variation, and acute parasitaemia which becomes chronic [22-26]. Also, as in *P. falciparum *infections, the inability to control the consequences of the first peak of parasitaemia is a major determinant of severe disease and death. In *P. chabaudi *and *P. falciparum *most investment into sexual stages occurs after the initial peaks of parasitaemia and commitment to gametocytes appears to follow changes in anaemia and red cell dynamics [27-30]. In addition, the bank of genetically distinct and phenotypically well-characterized clones available for *P. chabaudi *is substantially larger than for all other rodent malaria models [17].

The development of gene transformation technologies for *P. chabaudi *will, therefore, provide opportunities for experimental and analytical advances in fields as diverse as immunology and evolutionary ecology. This paper presents the first report of the generation of fluorescent *P. chabaudi *lines that constitutively express high levels of Green Fluorescent Protein (GFP) throughout their life-cycle.

## Methods

### Preparation of parasites for transfection

To obtain parasites for transfection, male MF1 mice (10 weeks) were infected *i.p*. with 1 × 10^7 ^*P. chabaudi *parasites from clone AJ4916. 500 μl of blood containing ring and early trophozoite-stage parasites were collected by cardiac puncture at 3 days post infection (5–10% parasitaemia). Parasites were cultured for 17–18 hours at a 1.5% dilution in complete culture medium (RPMI medium (with NaHCO3, Hepes and L-glutamine, Invitrogen) containing 25% heat inactivated foetal calf serum (Gibco), at pH 7.25) in the presence of 10% O_2_, 5% CO_2_, 85% N_2_, at 32°C in upright 200 ml flasks (Ikawa) in a horizontal shaking incubator at 30 rpm. Parasitized blood forming a layer at the bottom of the flask was gently removed and centrifuged at 1500 rpm for 30 sec. 5 μl of pelleted cells (1 × 10^6 ^schizonts) were used per transfection.

### Transfection and selection of transformed parasites

*Gfp *was introduced into the genome of *P. chabaudi *parasites, using the PbGFP_CON _plasmid previously described [7]. 5 μg of plasmid DNA, linearized at the Apa1 site, in 5 μl of dH_2_O was added to 100 μl of Amaxa nucleofector^tm ^test solution 88A6 (Basic Parasite Nucleofector^tm ^solution 2) in the manufacturers' cuvette (Amaxa Biosystems). 5 μl of schizont mix was added and electroporation was carried out in an Amaxa nucleofector^tm ^using program U33. After electroporation, 50 μl of pre-warmed complete culture media was added and the transfection mix was immediately injected *i.v*. into an MF1 mouse. Recipient mice received 35 μg/ml pyrimethamine in their drinking water (pH 3.5–5) for seven days post infection; the minimum pyrimethamine dose required to clear *P. chabaudi *infections of clone AJ in 24 hours (J. Thompson, personal observation). Giemsa-stained smears were scanned every one to two days and parasites that produced patent infections (days 14–20) were immediately passaged to further mice for the production of stabilate stocks.

### Analysis of transformed parasites

For genetic analysis of the integration locus, DNA was isolated as previously described [2] and Southern blot and PCR analyses were performed. PCR amplifications were carried out using Qiagen Taq DNA polymerase in the presence of 'Q solution' under the following conditions; 35 cycles; anneal, 54°C, 1 min; extend, 68°C, 3 min, denature, 94°C, 10 sec. To demonstrate integration into a *ssu-rrna *locus at the 5' region, amplification was carried out with primer Pc5'F (TTGTAAGAACGTGCTTGGTG) that is specific for *P. chabaudi ssu-rrna *sequence on *P. chabaudi *genome contig827, in the target region, and primer Pl5'R (TTCCCAGTCACGACGTTG) that anneals to *P. berghei d-ssu rrna *sequence in the PbGFP_CON _plasmid. To demonstrate integration into the contig827 *ssu-rrna *locus at the 3' region, amplification was carried out with primer Pc3'R (AGAGCCCAGCGATGAC) that is specific for *P. chabaudi *contig827 *ssu-rrna *sequence in the integration site, and primer Pl3'F (CAATGATTCATAAATAGTTGGAC) that anneals to *P. berghei d-ssu rrna *sequence in the PbGFP_CON _plasmid. To demonstrate the presence of *tg-dhfr *sequence, amplification was carried out with primers L190 (CGGGATCCATGCATAAACCGGTGTGTC) + L191; CGGGATCCAAGCTTCTGTATTTCCG. To amplify circular PbGFP_CON _plasmid, amplification was carried out with primers PlF2 (AATCATGACTTCTGTCACTGC) and Pl5'R. Primers Pc5'R and Pc3'R anneal specifically to sequences within *P. chabaudi *contig827 *ssu-rrna *and not to sequences within the Pb_CON _vector. Probe template for the detection of *tgdhfr *by Southern blots was amplified using primers L190/L191. DNA was digested with HindIII and NheI, transferred to Hybond N^+ ^membrane (Amersham) and hybridized according to the manufacturers methods. Wet preparations of live parasites expressing GFP were visualized using Openlab digital imaging (Improvision). The development and progression of parasitaemia in transformed parasites was compared to the wild type ancestor by following six mice infected with 10^6 ^parasitized red blood cells for each line. Infections were monitored daily to collect red blood cell density and parasitaemia data until day 14 post infection, when the acute phase parasites had been cleared. The infection dynamics of the two lines were analysed using linear mixed-effects models, which account for repeated measures across infections. One mouse from each line was euthanized (day 10 and 11) so these infections did not contribute data for the whole time course.

## Results and discussion

The optimal stage for DNA uptake by *Plasmodium *is thought to be the free merozoites, released at schizogony, that are not surrounded by red blood cell cytoplasm and membranes.*P. berghei *may, therefore, be relatively amenable to genetic transformation because schizonts developing in reticulocytes do not rupture in *in vitro *culture conditions, so high numbers can be purified. These rupture, releasing merozoites, during electroporation. In contrast, *P. chabaudi *schizonts, developing in mature red blood cells, do not arrest in culture and cannot be purified in such large numbers. For this reason, the transformation efficiency of *P. chabaudi *is likely to be considerably lower that that of *P. berghei*. The major improvements in rodent malaria parasite transformation efficiency obtained with the Amaxa Nucleofector^tm ^technology [9], however, together with the development of culture conditions that generate mature *P. chabaudi *schizonts suggested to us that transformation of *P. chabaudi *may now be possible.

**Figure 1:**
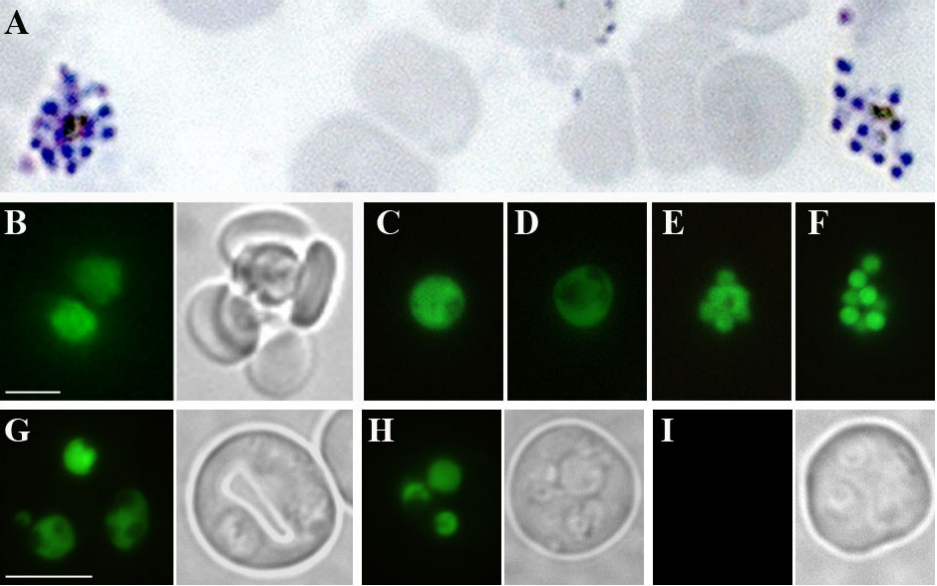
**(A) Cultured *P. chabaudi *schizonts used for transfection (giemsa stain) (B-F) Fluorescent and bright field images of *P. chabaudi *PcGFPcon blood-stage parasites**. (B) two trophozoites in a rosetting red blood cell; (C) young trophozoite; (D) gametocyte; (E) schizont; (F) rupturing schizont; multiple infection of young and mature trophozoites (G); multiple infection of rings and young trophozoite (H); wild-type AJ *P. chabaudi *(I). Scale bar = 5 μm.

**Figure 2:**
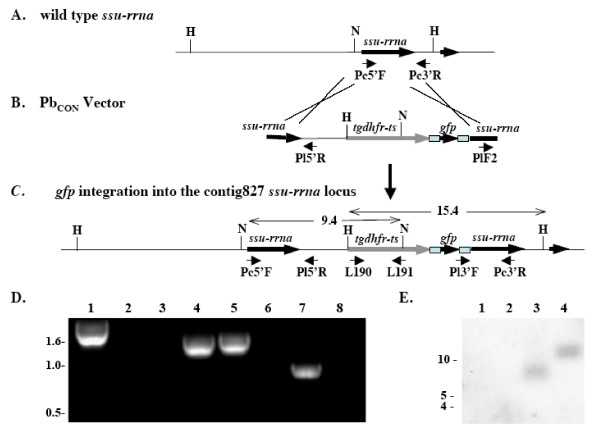
**Integration of Pb_CON _into *P. chabaudi ssu-rrna***. Schematic representation of *P. chabaudi ssu-rrna *locus on contig827 (A). Schematic representation of linearized plasmid Pb_CON _containing the Tgdhfr-ts cassette, conferring pyrimethamine resistance and *gfp*, flanked by *P. berghei d-ssu-rrna *target sequences for integration (B). Schematic representation of the contig827 *ssu-rrna *locus in *Pc*GFP_CON _clone 2.3, following integration of the Pb_CON _plasmid (C). D: *tgdhfr *is present in *Pc*GFP_CON _(lane 1) but not wt (lane 2) DNA; amplification with L190/L191. Circular PbGFP_CON _plasmid is absent in *Pc*GFP_CON _(lane 3); circular *Pb*GFP_CON _(lane 4); amplification with PlF2/Pl5'R. Verification of the 5' integration site; Pc5'F/Pl5'R amplify a product in *Pc*GFP_CON _(lane 5) but not in wt (lane 6) DNA. Verification of the 3' integration site; Pl3'F/Pc3'R amplify a product in *Pc*GFP_CON _(lane 7) but not wt (lane 8) DNA. E: *Tgdhfr *is present in the genome of *Pc*GFP_CON _but not wt parasites. wt (lanes 1 and 2) and *Pc*GFP_CON _(lanes 3 and 4) genomic DNA digested with NheI (lanes 1 and 3) or HindIII (lanes 2 and 4). Integration of *Pc*GFP into the *P. chabaudi ssu-rrna *locus on contig827 results in restriction enzyme digestion products of 9.4 and 15.4 kb following NheI and HindIII digestion respectively. N, NheI; H, HindIII.

To obtain appropriate numbers of *P. chabaudi *schizonts, the *in vitro *culture protocol described by Mackinnon *et al *[24] was adapted and scaled-up. Under these conditions, synchronous *P. chabaudi *ring-stage parasites/young trophozoites develop over 17–18 hours into a population of parasites that contain > 10% mature schizonts (Figure 1A). Schizonts produced by *in vitro *culture of *P. chabaudi *AJ blood stage parasites were transfected with the PbGFP_CON _plasmid [7] that contains an incomplete copy of the *P. berghei d-ssu-rrna *as a target region for integration, and the pyrimethamine-resistant *tgdhfr-ts *selectable cassette (*pyrR2*) for selection of transgenic parasites (Figure 2). Blast analysis of the *P. chabaudi *genome  showed that there is highest identity (94%) between the *P. berghei d-ssu-rrna *target region and *P. chabaudi ssu-rrna *sequences on genome contig827, indicating that these are orthologous loci. Linearized PbGFP_CON _DNA was introduced into the parasite genome by electroporation and recombinant parasites that express *tghfr *were selected by treatment with pyrimethamine.

Integration of the PbGFP_CON _cassette into the *P. chabaudi *genome was confirmed by PCR analysis in four independent lines and line '2.3' was selected for further genetic and phenotypic analysis. In *P. chabaudi *line 2.3, PbGFP_CON _integrated into the genome of contig 827 rRNA subunit, orthologous to the *P. berghei c *or *d-ssu-rrna *that have previously been shown to be non-essential genes in rodent malaria parasites [7]. Recombinant parasites develop to blood-stages with no apparent defects (Figure 1B–E) and form gametocytes that exflagellate and can infect mosquitoes.

**Figure 3:**
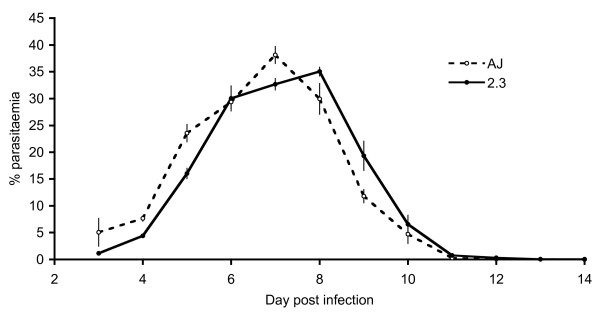
**Blood stage dynamics of wild type and *Pc*GFP_CON _parasites**. Average (± standard error) parasitaemia of six infections with *Pc*GFP_CON _(2.3) and wild type AJ (AJ4916) parasites. Infections were initiated with 10^6 ^parasitized red blood cells and followed by microscopy throughout the acute phase.

The *in vivo *asexual dynamics of line 2.3 was not significantly different to wild type *P. chabaudi *AJ4916 ancestral parasites that had undergone comparable numbers of passages (Figure 3). Infections initiated with line 2.3 and AJ4916 parasites both reached patency by microscopy on day 3 post infection, peaked on day 7–8 and the acute phase lasted for 14 days. More detailed analysis of parasiteamia revealed that the lines did not differ significantly in their average parasitaemia (F_(1,10)_= 0.02; P = 0.901). The patterns observed throughout infections were similar, though parasitaemia of line AJ4916 was significantly lower on days 8 and 9 post infection (F_(10,91) _= 4.96; P < 0.0001). The lines did not differ in the patterns or the average levels of anaemia they caused (F_(1,10) _= 0.001; P = 0.975). Although reversion to the wild-type genotype was observed at a low rate after multiple blood-stage passages, all Pc-GFP_CON _(line 2.3) parasites observed at day 8 of infection were GFP-positive.

## Conclusion

*Plasmodium chabaudi *is reproducibly accessible for genetic transformation at an efficiency that is sufficient for genomic integration of introduced genes. The development of technologies that allow disruption or modification of gene expression in *P. chabaudi*, thus, opens the way for direct functional analysis of parasite proteins throughout both acute and chronic stages of an *in vivo *malaria infection, including those that have been implicated in modulation of the host immune response [31]. The generation of stable fluorescent *P. chabaudi *parasite lines also offers the opportunity for imaging of direct interactions between the parasite and host cells within a variety of host tissues.

## Abbreviations

GFP: green fluorescent protein; Tgdhfr: Toxoplasma gondii Dihydrofolate reductase.

## Competing interests

The authors declare that they have no competing interests.

## Authors' contributions

Both authors conceived and designed the project and prepared the manuscript. SR prepared and characterized parasites and JT undertook the transfection and molecular analyses. All authors read and approved the final manuscript.
